# Rethinking Norm Psychology

**DOI:** 10.1177/17456916221112075

**Published:** 2023-07-13

**Authors:** Cecilia Heyes

**Affiliations:** Department of Experimental Psychology & All Souls College, University of Oxford

**Keywords:** cognitive gadgets, human cooperation, cultural evolution, domain-general learning, economic games, evolutionary psychology, moral psychology, norm psychology, reinforcement learning, social learning

## Abstract

Norms permeate human life. Most of people’s activities can be characterized by rules about what is appropriate, allowed, required, or forbidden—rules that are crucial in making people hyper-cooperative animals. In this article, I examine the current cognitive-evolutionary account of “norm psychology” and propose an alternative that is better supported by evidence and better placed to promote interdisciplinary dialogue. The incumbent theory focuses on rules and claims that humans genetically inherit cognitive and motivational mechanisms specialized for processing these rules. The cultural-evolutionary alternative defines normativity in relation to behavior—compliance, enforcement, and commentary—and suggests that it depends on implicit and explicit processes. The implicit processes are genetically inherited and domain-general; rather than being specialized for normativity, they do many jobs in many species. The explicit processes are culturally inherited and domain-specific; they are constructed from mentalizing and reasoning by social interaction in childhood. The cultural-evolutionary, or “cognitive gadget,” perspective suggests that people alive today—parents, educators, elders, politicians, lawyers—have more responsibility for sustaining normativity than the nativist view implies. People’s actions not only shape and transmit the rules, but they also create in each new generation mental processes that can grasp the rules and put them into action.

Human lives are drenched in social norms. People’s clothes, eating habits, sexual and parental behaviors, and day-to-day modes of interaction with one another—from greeting and speaking to helping and harming—can be described by rules about what is appropriate, allowed, required, or forbidden in different contexts for various members of a social group. Some norms are crisply codified in law (e.g., drive on the right, thou shalt not kill), whereas others would be difficult for any group member to articulate (e.g., how much eye contact is appropriate in conversation with superiors, subordinates, equals). Some have a moral flavor—a prohibition against unnecessary harm to other people may apply to everyone at all times—whereas other norms, such as who should wear a particular kind of hat, are obviously transitory and group-specific. Norms vary on many dimensions, but norms of some sort appear to be present in all human cultures (Brown, 1991) and to be rare, minimal, or absent in other animals (Jensen, 2016; Schmidt & Rakoczy, in press; for contrasting views, see Andrews, 2020; Fitzpatrick, 2020).

“Norms” have been part of the conceptual tool kit of anthropology, economics, politics, sociology, and social psychology for as long as those disciplines have existed. “Norm psychology” emerged more recently and has a different fan base. About 15 years ago, scholars interested in human evolution—anthropologists, biologists, economists, philosophers, and (a few) psychologists—began to use “norm psychology” (or “normative cognition”) to refer to a set of cognitive and motivational mechanisms that, they believe, have been specialized by genetic evolution for processing social “rules” or “behavioral standards” (e.g., Boyd & Richerson, 2005; Chudek & Henrich, 2011; Fehr & Schurtenberger, 2018; Fitzpatrick, 2020; Henrich, 2020; Henrich & Muthukrishna, 2021; House, 2018; House et al., 2020; Kelly & Davis, 2018; Mikhail, 2011; O’Neill & Machery, 2018; Richerson et al., 2016; Sripada & Stich, 2006; Zefferman, 2014). “Norm psychologists,” whatever their disciplinary affiliation, believe that the mental processes guiding normative behavior are important, domain-specific, and genetically inherited. They are important because norms enable cooperation, and the human capacity for cooperation is a large part of what makes people such peculiar, and peculiarly successful, animals. They are domain-specific in the sense of being different from the cognitive and motivational mechanisms that do other jobs in the mental economy, such as predicting feeding opportunities or detecting predators. And the domain-specific features of norm psychology, rather than basic ingredients found in other animals, are programmed in people’s genes. According to the gene–culture (or culture–gene) coevolutionary view supported by norm psychologists, norm content—for example, the prescription or proscription of cousin marriage or hat wearing—is learned through social interaction. However, during human evolution, the effects on behavior of norm content provided powerful selection pressure for the genetic evolution of psychological mechanisms specialized for norm processing. On this account, norm content is acquired and implemented by mechanisms that have been tailored for norm processing by natural selection acting on genetic variants.

The first of these claims, about the importance of norm psychology, is rock solid. Questions remain about exactly what types of norms allowed early humans ancestors to begin cooperating with unrelated others and, ultimately, in some societies to cooperate over long timescales and at high risk—for example, in financial markets (Boyd, 2016; Boyd et al., 2003; Boyd & Richerson, 2001; Henrich, 2015; Sterelny, 2021)—but no one would deny that norms of some kind were and are crucial for human cooperation. Given their importance, norms need to be explained not only functionally, in terms of their effects on behavior and fitness, but also at the internal, psychological level. Researchers need to know what it is about human minds that enables them to learn, implement, and enforce norms and where those features come from. Without this information, the understanding of norms is radically incomplete; there is a missing link between the social science and the natural science of norms, and researchers have limited capacity to inform business, education, and government in designing laws and policy interventions to promote cooperation in contemporary societies (Kelly & Morar, 2020; Raymond et al., 2021).

It is precisely because norm psychology is important that the other claims of norm psychologists, about domain-specificity and genetic inheritance, deserve closer scrutiny. The domain-specificity claim is contrary to theories of norm processing rooted in social psychology (Gross & Vostroknutov, 2022), philosophy (Bicchieri & McNally, 2018; Colombo, 2014; Nichols, 2021), and cognitive neuroscience (Theriault et al., 2021; Veissière et al., 2019), but the two camps—norm psychologists and domain-generalists—rarely talk to one another. Norm psychologists cite evidence they regard as consistent with domain-specificity and genetic inheritance but do not address the evidence interpreted by domain-generalists to show that normative behavior depends on cognitive and motivational processes that do many other jobs. Likewise, if they refer to the work of norm psychologists at all, domain-generalists usually just state their disagreement. No one is testing the theories against one another, assessing in a systematic, scientifically healthy way to what extent the evidence favors domain-specificity over domain-generality or vice versa (Press et al., 2022).

The standoff may be due to a sense on both sides that they have different purposes. Norm psychologists want to understand human evolution, whereas domain-generalists have a tighter focus on the character and development of normative thinking and behavior in contemporary Western societies. This contrast does not justify a lack of engagement or make the standoff less wasteful of intellectual and financial resources. There are clearly areas of common purpose in which the two camps are making conflicting claims that need to be resolved. But it is not unusual in academia and everyday life for people with different projects and backgrounds to live in bubbles.

The overriding purpose of this article is to burst the normativity bubbles, to draw norm psychologists and domain-generalists together for productive debate in future research on the psychology of normativity. I aim to do this by outlining a cultural evolutionary account of norm psychology. This account proposes that, in humans, normative competence depends on domain-general psychological processes plus a culturally evolved “cognitive gadget” (Heyes, 2018a, 2019b). A cognitive gadget is a distinctively human, domain-specific cognitive process (or integrated set of cognitive processes) that is assembled through social interaction during childhood. At the population level, cognitive gadgets are shaped to do their jobs by cultural selection. Cultural selection is a Darwinian process of variation and selective retention operating on socially inherited, rather than genetically inherited, variants (Birch, 2017; Birch & Heyes, 2021; Campbell, 1965; Lewens, 2015). Thus, like the “cognitive instincts” postulated by evolutionary psychologists in the 1990s (Pinker, 1995), cognitive gadgets are adaptations—products of a Darwinian selection process—but they are predominantly social rather than genetic adaptations.^1^

The gadget account is synthetic in being both “cognitive-evolutionary,” like the nativist view (Kelly & Setman, 2021), and compatible with the evidence that domain-general processes are important in the development of normative behavior. I argue that it fits current evidence better than previous evolutionary accounts, but my purpose is not to show that the gadget account is *right*. Not surprisingly, I think it has many merits, but the function of this article is to provide a framework for future research in which conflicting claims can be tested against one another. To this end, I propose not only an alternative cognitive-evolutionary theory of normativity but also a new “poverty-wealth scheme” for testing it against the original.

I begin by summarizing the tenets of “nativist norm psychology,” the evolutionary framework that the gadget account seeks to revise. I then discuss problems with the nativist view that motivate revision and present the cultural-evolutionary alternative. The final section discusses some potential objections to the cultural-evolutionary framework and its implications for research and in the wider world.

## Nativist Norm Psychology

Norm psychology descends from and overlaps with “moral psychology,” a project also pursued collaboratively by philosophers and scientists. Norm psychology is broader than moral psychology in its concern with “conventional” norms (e.g., relating to duration of eye contact) as well as “injunctive” or “prescriptive” norms (e.g., relating to harm) and narrower in focusing on norm acquisition and implementation. Unlike moral psychology, norm psychology is concerned with the psychological processes mediating altruism, well-being, character, virtue, and the moral emotions, such as shame and guilt, only insofar as they influence an individual’s capacity to acquire and implement social rules (Kelly & Setman, 2021). Norm psychology was born out of discontent with moral psychology (Westra & Andrews, in press). However, the overlap remains so substantial that, in this article, I often refer to the work of moral psychologists (e.g., Bear & Knobe, 2017; Cushman, 2013; Greene, 2017; Haidt, 2001, 2012). I also refer to important, independent empirical work by Tomasello and colleagues (e.g., Göckeritz et al., 2014; Schmidt et al., 2019). Unlike norm psychology, the theory they have developed depends on a constructivist rather than a computational view of the mind.

Sripada and Stich (2006) coined the term “norm psychology” and provided a compelling manifesto in a chapter titled “A Framework for the Psychology of Norms.” They gave (a) a “characterisation” of norms, (b) a survey of evidence that norm psychology is “innate,”^2^ and (c) a preliminary model of the computational mechanisms enabling the acquisition and implementation of norms. The following summary of nativist norm psychology uses their framework as a springboard because, even now, it captures the key tenets more clearly than any other statement I have found. In addition, crucially, it remains representative of the field. With minor exceptions, which I note, most nativist norm psychologists work within Sripada and Stich’s framework. They use similar definitions, the same categories of evidence, and in many cases, the same key examples.

### Characterization: rules

A norm is “a rule or principle that specifies actions which are required, permissible or forbidden” and has “independent normativity” and “intrinsic motivation” (Sripada & Stich, 2006, p. 281). Independent normativity means that to qualify as a norm, a rule may be, but need not be, recognized and enforced by social institutions and laws. Intrinsic motivation means that “people are motivated to comply with norms as *ultimate ends*, rather than as a means to other ends” (Sripada & Stich, 2006, p. 281; see also Gavrilets & Richerson, 2017; Henrich & Ensminger, 2014). “Violations of norms, when they become known, typically engender *punitive attitudes*, like anger, condemnation, and blame, directed at the norm violator, and these attitudes sometimes lead to punitive behaviour” (Sripada & Stich, 2006, p. 281).

### Evidence of innateness

The empirical case for the innateness of norm psychology can be summarized in four propositions relating to universality, importance, development, and motivation.

#### Norms are universal

There is a broad consensus that norms are present in all human cultures and that although there are common themes, norm content is highly diverse (Brown, 1991; Henrich, 2020; Wilson & Sober, 1998). Because of this combination of commonality and diversity, norm psychologists tend to be agnostic about whether norm content is innate. They leave open the question of whether humans are born with a genetically inherited propensity to believe that specific behaviors are prescribed or forbidden. However, they assert that norm content is acquired and implemented by innate psychological mechanisms, cognitive and motivational processes that have been tailored for norm processing by natural selection acting on genetic variants.

The combination of commonality and diversity is obvious in some domains, such as clothing and body adornment. Most societies have sartorial norms, but the prescribed and proscribed items vary from penis sheaths to powdered wigs. Less obviously, the commonality-plus-diversity pattern is present in domains more closely associated with cooperation and morality. Most societies have norms prohibiting killing and physical assault and promoting sharing, reciprocation, and helping, but there is wide variation in tolerance of harmful behavior, especially against women and out-group members, and in the extent of helping expected in various contexts and by people in different social roles.

#### Norms are important

Sripada and Stich (2006) emphasized, uncontroversially, that norms were important in human evolution and that they continue to play crucial roles in contemporary life. Norms

govern a vast array of activities, ranging from worship to appropriate dress to disposing of the dead. And while some norms deal with matters that seem to be of little importance, others regulate matters like status, mate choice, food and sex that have a direct impact on people’s welfare and their reproductive success. (Sripada & Stich, 2006, p. 282)

#### Normative behavior develops early and without teaching

There is plenty of evidence that normative behavior appears early in childhood. Sripada and Stich (2006) cited evidence that children can distinguish prescriptive from descriptive social rules at 3 to 5 years of age (Nucci, 2001) and that cross-cultural variation in fairness norms is in place by 9 years of age (Henrich et al., 2004). Since 2006, developmental studies have revealed other precocious normative achievements. For example, 5-year-olds are not only more likely to help family members than friends or strangers (“kin favouritism”; Lu & Chang, 2016) and to help people who have helped them (“direct reciprocity”; Warneken & Tomasello, 2013)—behaviors that are not thought to require domain-specific cognition—but they are also more likely to help individuals who were previously observed helping rather than harming a third party (“indirect reciprocity”; Li & Tomasello, 2018). Yet more striking, 6-year-olds punish unequal distribution of resources between third parties when delivering punishment incurs a cost (McAuliffe et al., 2015), and 3-year-olds engage in “normative protest,” saying things such as “No, it does not go like this!” when a puppet fails to do the same thing as an adult (Kenward, 2012; Rakoczy et al., 2008; Schmidt & Rakoczy, in press).

#### Normative behavior is intrinsically motivated

Normative behavior is intrinsically motivated in the sense that norms have a “unique kind of subjective authority which differs from standard instrumental motivation” that makes people “disposed to comply with norms even when there is little prospect for instrumental gain, future reciprocation or enhanced reputation, and when the chance of being detected for failing to comply with the norm is very small” (Sripada & Stich, 2006, p. 285). This view, that has been standard in moral philosophy, sociology (Durkheim, 1912/1968), and parts of social psychology (Batson, 2014) for many years, is supported by everyday examples such as tipping in a restaurant to which you know you will never return and jumping in a river to save a drowning person (Frank, 1988). For hard data, norm psychologists turn to behavioral economics, highlighting evidence that, in experimental games, people cooperate—for example, give more money than necessary to another player—when players are anonymous and aware that they will have just one exchange (Marwell & Ames, 1981). People also punish norm violation—for example, failure to contribute to common goods—when all players are anonymous and delivering punishment incurs a cost (Fehr & Gächter, 2002). This kind of “costly punishment” of “free-riders” occurs both when punishers have lost out because of norm violation, and, in the case of “third party punishment,” when they have merely observed norm violation in a game in which the punisher had no stake (Fehr & Fischbacher, 2004).

### Model

**Fig. 1.**
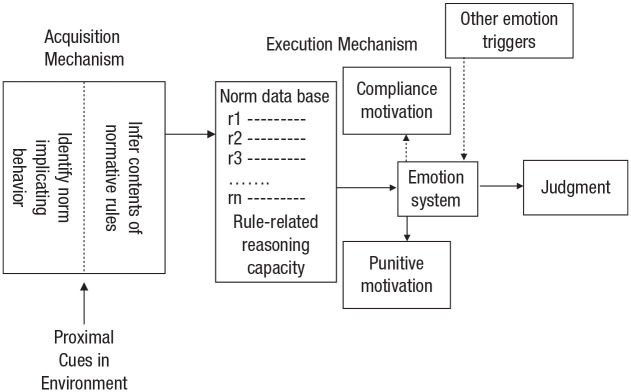
Sripada and Stich’s (2006) “sketch of the mechanisms underlying the acquisition and implementation of norms” (redrawn from Sripada & Stich, 2006, p. 298).

Figure 1 shows Sripada and Stich’s (2006) model. The authors suggested that both main components, the acquisition mechanism and the execution mechanism, are innate and domain-specific; they have been shaped by genetic evolution to operate in a different way from mechanisms that acquire and implement rules that are not norms. The acquisition mechanism operates automatically from early in development. It detects behavioral cues indicating that there is a norm in the local cultural environment, infers the content of that norm, and passes this information about norm content to the implementation system, where it is stored and used. The implementation mechanism maintains “a data base of normative rules,” supplied by the acquisition mechanism; generates intrinsic motivation to comply with those rules; detects violations; and generates intrinsic motivation to punish violators.

Elaborating on this sketch, Sripada and Stich (2006) suggested that some norms are easier than others to detect, infer, remember, and/or recall because the acquisition mechanism is constrained by innate biases that are specific to social learning—learning from others, rather than direct interaction with the inanimate environment. The candidates here are “Sperberian biases”—preferences, aversions, and emotions that make some ideas more “attractive” than others (Sperber, 1996)—and “social learning strategies” that incline people to learn more from older, more prestigious models (“prestige bias”) or to adopt the most common cultural variant (“conformity bias”; Henrich & Boyd, 1998; Henrich & Gil-White, 2001; for a recent critique of conformity bias, see Lewens, 2015).

Sripada and Stich (2006) assumed innate motivation to punish norm violation, driven by anger, contempt, and disgust, but, like subsequent norm psychologists, stopped short of claiming there are norm-specific emotions (Kelly, 2020a). Their model suggests that beliefs, judgment, and explicit reasoning are parts of a semidetached system—what I call the “explicit system”—shown on the right side of Figure 1.^3^

## Problems With the Nativist View

Sripada and Stich’s (2006) framework gave norm psychology a strong start, but its use in the last 15 years and independent developments have highlighted conceptual and empirical problems.

### Explanatory target

Sripada and Stich (2006) defined norms as “rules” and norm psychology as a system for processing these rules. This is troublesome because it is not clear what distinguishes a mental representation of a rule (or a rule-like mental representation) from any other kind of mental representation. Paradigmatic examples of rules are written or spoken statements such as “Thou shalt not kill” or “Drive on the right”; they are carved in stone, a computer, or the airwaves, not in the head. If one takes the paradigmatic cases to mean that mental rules are sentences in the head, then a rule-based definition of norms presupposes what research on norm psychology is intended to discover—the nature of the psychological processes underlying normative behavior. Instead of encouraging open inquiry about the kinds of mental representations involved in norm processing, it presupposes that normative behavior is mediated by complex, language-like mental representations. If, like Sripada and Stich, researchers avoid this assumption, a rule-based definition of norms leaves the field deeply uncertain about the explanatory target of norm psychology (Westra & Andrews, in press). What is the nature of a rule when it is *not* written, spoken, or inscribed in the mind in sentential form?

This problem has not been ironed out since Sripada and Stich’s (2006) seminal work was published. Following their lead, philosophers pursuing a cognitive-evolutionary approach have offered detailed, rule-based characterizations of norms (e.g., Kelly & Setman, 2021). Norm psychologists from other disciplines either do not define norms or norm psychology at all (e.g., Henrich & Muthukrishna, 2021), adopt a rule-based definition (e.g., “customary rules that govern behavior in groups or societies”; House, 2018), or, without explanation or commentary, replace “rules” with “standards.” For example, norms have been defined as “learned behavioral standards shared and enforced by a community” (Chudek & Henrich, 2011, p. 218) and as “mutually accepted behavioral standards of a group” (Göckeritz et al., 2014). The term “standard” has the advantage of not implying a particular format of mental representation, but it raises other troublesome questions. For example, in the context of norm psychology, is a standard a level of quality or merely what is common? To be counted as a normative agent, do individuals have to represent standards, or is it sufficient for their behavior to conform with the standards? Does normative competence require that an individual knows the standard is “shared” or “mutually accepted”? If so, how is that knowledge represented? Thus, even when “rules” are replaced by “standards,” the explanatory target of norm psychology remains obscure.

### Evidential relations

Norm psychologists typically do not explain for each category of evidence—universality, importance, early development, intrinsic motivation—why they take it to support the existence of innate, domain-specific mechanisms for norm processing. Sripada and Stich (2006) simply stated “it is hard to see how the facts we’ve assembled *could* be explained without positing innate psychological mechanisms that perform the functions we’ve sketched” (pp. 290–291). This is problematic because the evidential relations are far from obvious.

#### Universality

The mere presence of norms in all human societies does not imply that norms are acquired and implemented by norm-specific, genetically inherited mechanisms. In principle, normativity could be due to convergence. All human societies, confronting similar problems of cooperation and with a common kit of domain-general psychological processes, may have found roughly the same solutions (Buckholtz & Marois, 2012). This would be implausible if all societies had found exactly, rather than roughly, the same solutions. It would be implausible if norm content were universal—for example, with all societies forbidding cousin marriage and violence against women and having the same fairness norms—but norm psychologists freely acknowledge that this is not the case. It would also be implausible if norms were equally important in all societies, but research has revealed marked cross-cultural variation in the “tightness” of social norms. Tight cultures (e.g., India, Japan) have many, strict norms and low tolerance of deviant behavior, whereas loose cultures (e.g., the Netherlands, Ukraine) have fewer, weaker norms and greater tolerance of deviant behavior (Gelfand, 2018; Gelfand et al., 2011). Variation in tightness does not preclude innate norm psychology, but it does highlight the difficulty of making inferences from cross-cultural data to psychological mechanisms. It is not clear how much norm variation, and of what kind, would favor an empiricist over a nativist view of norm psychology or vice versa (Sterelny, 2010).

#### Importance

Gelfand (2018) and Gelfand et al. (2011) work on tightness suggests that norms are less important in some societies than in others. This does not undermine the general claim that normativity is an important feature of human lives, but it raises the question whether the importance of norms should incline researchers to believe that norm-processing mechanisms are innate. Reading and writing are important in many societies, but because of their recent historical origin, we know that reading and writing are not mediated by dedicated, innate mechanisms. Human characteristics can be pervasive and important without being genetically inherited. So perhaps the inference from importance to innateness depends on the observation that some norms “regulate matters like status, mate choice, food and sex that have a direct impact on people’s reproductive success” (Sripada & Stich, 2006, p. 282). If so, the inference remains cryptic. If norms have an impact on reproductive fitness, there is the potential for *something* related to their processing to be favored by genetic evolution, but that something need not be “Big Special” psychological processes—complex, uniquely human mechanisms dedicated to processing a specific kind of input. Instead, any norm-related genetic adaptations could be “Small Ordinary”—simple, quantitative modifications of mechanisms present in other animals (Heyes, 2018a, 2019b).

#### Early development

Chomsky (1965) made a compelling “poverty of the stimulus” argument linking development with innateness. Focusing on language, he argued that there is reason to believe a characteristic is innate—that a specific propensity to develop the characteristic is genetically inherited—when it appears before children have been exposed to enough information in their environment to support development of the characteristic. For example, there would be reason to believe in an innate “language acquisition device” if children’s linguistic development runs ahead of the information about language provided by their social interactions with adults.

Whatever its merits for language in particular, the poverty argument provides a solid, general basis for inferring genetic inheritance from developmental data (Heyes, 2018a, 2019a). However, like subsequent norm psychologists (and unlike some moral psychologists, e.g., Hauser, 2006), Sripada and Stich (2006) did not advance poverty arguments. For example, they did not consider whether there is enough information in the developmental environment for children to learn, via domain-general mechanisms, to distinguish prescriptive from descriptive rules before they are 3 to 5 years old (Nucci, 2001). They cited ethnographic evidence that it is not necessary for children to be taught to punish norm violation—a point amplified by more recent experimental work with Western children (e.g., Schmidt & Rakoczy, in press)—but, viewed as a poverty argument, this is not compelling for two reasons. First, teaching is just one way in which information can be provided through social interaction, one potential contribution to “wealth” rather than “poverty” of the stimulus (Ray & Heyes, 2011). Adults, and other experts, can impart information about norms and normativity without intending to do so, “leaking” their attitudes in emotionally charged behavior and casual remarks about the actions of others (Heyes, 2019a). For example, in conversation with their children over a picture book, Western parents refer more often to fairness when explaining why in-group, rather than out-group, members should be helped and not harmed (Chalik & Rhodes, 2015). Second, whatever may or may not be necessary to support normative behavior in the laboratory, there is ample anecdotal and ethnographic evidence that in the wild, normative behavior, such as sharing and helping, are taught in infancy and learned through social play (Lew-Levy et al., 2018). This evidence suggests wealth, rather than poverty, of the stimulus—that there may be sufficient information in children’s environments to support the development of normativity. More generally, it reminds one that early development of a characteristic is not, by itself, evidence of innateness.

#### Intrinsic motivation

Sripada and Stich’s (2006) claim about intrinsic motivation was clear. In their view, intrinsic motivation—evidenced by selfless acts in everyday life and delivery of costly punishment in the lab—could be produced by norm-specific, genetically inherited mechanisms but not by domain-general processes. The claim was clear, but there was little argument. For example, like subsequent norm psychologists, they did not explain why, in their view, costly punishment could not be due to mistaken beliefs about the likelihood of future interactions with the same agents, generalization from the rewarding effects of selfish punishment, or domain-general, rather than norm-specific, social learning (Seymour et al., 2007). Likewise, norm psychologists have not explained why one should expect motivation from these sources to be less effective than motivation arising from domain-specific, genetically inherited processes in converting normative thought into action (Hume, 1748/1975).

In the last 15 years, additional evidence has been added to each of Sripada and Stich’s (2006) categories, but the evidential relations remain unclear. No one has spelled out why evidence of universality, importance, early development, and intrinsic motivation—independently or in combination—should encourage one to believe that humans have an innate norm psychology rather than normative competence based on domain-general cognitive processes and cultural learning.

### Counterevidence

Sripada and Stich’s (2006) model remains a high point in the history of norm psychology. As far as I am aware, it is the only attempt to date to specify the mechanisms involved in norm processing, and—aside from questions about rules and evidential relations (above)—the specification was clear and plausible in the light of evidence available at the time. However, subsequent empirical and theoretical developments have challenged key features of the model.

#### Common is right

A substantial body of evidence now shows that children and adults conflate what is descriptively normal (common or frequent) with what is prescriptively normal (allowed or required; Bear & Knobe, 2017; Eriksson et al., 2021; Foster-Hanson et al., 2021; McAuliffe et al., 2017; Roberts et al., 2018, 2019). For example, after being told that listening to a certain kind of music is common within a group, children between 4 and 13 years express disapproval of a group member who listens to a different kind of music and explain their disapproval using prescriptive language (e.g., “that’s not allowed”; Roberts et al., 2017). When adults from 30 European countries were asked about questionable behaviors (e.g., casual sex, paying cash to avoid taxes), there was a positive correlation between their ratings of the frequency and justifiability of the behavior (Eriksson et al., 2021). And the evidence of descriptive-prescriptive conflation is not only correlational. Adults learning a prescriptive norm from scratch, the ideal length of a fictional hunting tool called a “stagnar,” blended statistical and evaluative information in the training set. After learning, their representation of a “normal” stagnar depended on the distribution of stagnar lengths to which they were exposed during training (descriptive) as well as information given with each exemplar about “how good that stagnar is for hunting” (prescriptive; Bear & Knobe, 2017, Study 4).

This evidence of conflation does not sit well with the assumption that prescriptive norms—the focus of norm psychology—are acquired and implemented by mechanisms dedicated to processing prescriptive norms. Sripada and Stich’s (2006) model suggests that the acquisition mechanism is switched on by prescriptively normative behavior and infers norms from this behavior and that the products of these inferences are the only entries in the execution mechanism’s “norm data base.” If this were the case, one might expect statistical information—information about what is average, or common, rather than what is approved or ideal—to influence very young children or the speeded responses of adults when the cognitive system is immature or under heavy demand, but judgments about what is allowed or justifiable should not be pervasively skewed by this information throughout life.

To accommodate evidence of prescriptive-descriptive conflation, a nativist norm psychologist might argue that in ancestral environments, there were conditions in which it was adaptive for the norm-acquisition mechanism to overshoot, making false positive judgments. However, without specification of what these conditions were and evidence that they correspond with those in which contemporary actors “mistake” descriptive for prescriptive norms, this would be a fudge (Lipton, 2003). Alternatively, a norm psychologist might take prescriptive-descriptive conflation as a sign that domain-specific norm-acquisition mechanisms make use of innate social-learning strategies, such as conformist bias. Because conformist bias is thought to influence all social learning, this would be a significant retreat from the claim that norm processing depends on dedicated mechanisms. Given evidence that distinctively human social-learning strategies are culturally inherited (see below), it would also run contrary to the claim that norm psychology is innate.

In sum, the pervasive influence of descriptive information on normative judgment suggests either that prescriptive norm processing is not mediated by innate, domain-specific mechanisms or that such mechanisms exist but do not function well because their operation is regularly contaminated by information from outside their proper domain (Andreoni et al., 2021).

#### Domain-general learning is important

There is evidence that domain-general processes, especially reinforcement learning, play a major role in the development of normativity. For example, recent work on descriptive-prescriptive conflation shows that children and adults disapprove of atypical behavior not only in other people (relative to social categories) but also in nonhuman animals (relative to biological categories; Foster-Hanson et al., 2021). Likewise, studies of typically developing adults in multiplayer games indicate that their learning of new norms from robot players is subject to the same biases as learning character traits from observable behavior (a social but not normative task; Hertz, 2021).

A range of formal and informal models show that reinforcement learning would be an efficient way to acquire norms (Buckholtz & Marois, 2012; Ho et al., 2017; Morris & Cushman, 2018). Some of the empirical evidence implicating reinforcement learning comes from studies in which adults are asked to evaluate each of a series of stimuli (e.g., the attractiveness of faces or pieces of music) and told after each judgment whether it agreed with other people’s evaluations. Information indicating agreement activates the brain’s reward system, and information indicating disagreement activates areas processing punishment. In other words, a signal indicating to people that their behavior was or was not normative activates the same neural mechanisms as delivery of food or money (reward) or electric shock (punishment) for button pressing in response to inanimate stimuli (Germar & Mojzisch, 2019; Klucharev et al., 2009; Schnuerch & Gibbons, 2014; Wu et al., 2016). Increasing the resolution of these neurophysiological results, a recent study showed that learning about normative responses (which of two options is preferred by a social group) and about the inanimate world (points associated with selecting one of two boxes) is mediated by the same dopamine-dependent neurochemical mechanisms (Rybicki et al., 2022).

A nativist norm psychologist might object that the foregoing studies relate to descriptive rather than prescriptive norms, for example, to preferences that happen to be typical of a group but that are not explicitly endorsed by group members as the right preferences to have. The findings reviewed above suggest that prescriptive norms are not psychologically distinct from descriptive norms. However, for people who are not persuaded by descriptive-prescriptive conflation, there is further evidence of domain-general processing in which the subject matter is squarely prescriptive or even moral. For example, psychopaths—people with a developmental disorder that interferes with moral judgment and increases the risk of antisocial behavior—show impaired domain-general learning (Blair, 2017). Likewise, a study using electrophysiological and behavioral measures confirmed that infants are more likely to approach an agent categorized by adults as “helping” rather than “hindering” (e.g., Hamlin, 2015; Hamlin et al., 2010). However, it also indicated that this preference for prosocial actors is due to the same neural mechanisms that mediate approach to and avoidance of inanimate objects (Cowell & Decety, 2015; Decety & Yoder, 2017). Consistent with these data, connectionist modeling indicates that the helper-hinderer effect in infants could be due to domain-general associative learning (Benton & Lapan, 2021; Heyes, 2019a; Scarf et al., 2012).

Nichols and colleagues have focused most consistently on unequivocally prescriptive norms (Nichols, 2021; Nichols et al., 2016; Partington et al., 2021). Answering “moral nativists” (Hauser, 2006; Levine et al., 2018) and using Bayesian modeling alongside behavioral experiments, they have shown that complex features of moral normativity could be due to domain-general processing—for example, tendencies to interpret rules as act-based rather than consequence-based, to assume that it is permissible to do things that are not explicitly prohibited, and to respond differentially to the violation of moral and conventional norms (Nichols, 2021). This “rational learning” model of morality assumes rich, genetically inherited cognitive resources—including the concepts of agent, intention, and cause—while making a powerful case that specifically moral concepts are acquired via domain-general processes of learning.

#### Social-learning biases are innate or domain-specific

Recent work does not accord with Sripada and Stich’s (2006) suggestion that Sperberian biases (Sperber, 1996) and social learning strategies provide an opportunity for genetically inherited domain-specific psychological mechanisms to bias norm learning.

Sperberian biases have proved difficult to investigate empirically because they comprise a mixture of weakly specified learning, motivational, and ecological factors (Buskell, 2017). Social-learning biases are more empirically tractable, and research in the last 15 years has indicated the kinds of models that children, adults, and nonhuman animals are most likely to copy (Kendal et al., 2018). However, this research suggests that social-learning biases are either innate and domain-general or culturally learned and domain-specific; they are not, as Sripada and Stich (2006) supposed, innate and domain-specific (Heyes, 2016a, 2016c).

Consider prestige bias as an example. There is evidence that chimpanzees (Horner et al., 2010), children (Chudek et al., 2012; Fusaro & Harris, 2008; McGuigan, 2013), and adults (Henrich & Henrich, 2010) are more inclined to copy high-status models, or models to whom other agents attend (there is some ambiguity about the meaning of “prestige”; Chellappoo, 2021), but careful scrutiny of these studies suggests that the prestige biases of animals and children up to about 5 years old could be due to phylogenetically ancient, domain-general attentional processes (Heyes, 2016b, 2017a, 2017b; Heyes & Pearce, 2015). In the simplest cases, the prestigious models are more likely to be copied because they are salient—bigger, more vocal, more likely than others to be found by gaze following. In contrast, at least some prestige bias in adulthood is due to domain-specific, deliberative, culturally inherited metacognitive processes (Heyes, 2016c; Heyes et al., 2020).^4^

#### Costly punishment is rare and old

A central feature of nativist norm psychology is the idea that humans are intrinsically motivated to punish norm violation. Inflicting harm on a norm violator, exacting “retribution,” is an end in itself. In support of this view, Sripada and Stich (2006) cited evidence from economic games showing that people are willing to pay a price to punish norm violators both when the violation reduces the payoff of the punisher (costly second-party punishment; Fehr & Gächter, 2002) and when it reduces the payoffs of other players (costly third-party punishment; Fehr & Fischbacher, 2004), that is, when the punisher merely observes the violation. Since 2006, laboratory research using economic games has provided further evidence of costly second- and third-party punishment in adults (e.g., Crockett et al., 2014) and children from 6 years of age (Marshall et al., 2021; McAuliffe et al., 2015). It has also indicated that retribution is a sufficient motive. For example, in the laboratory, adults engage in costly punishment even when it is “hidden”—that is, the punisher believes that the violator will not be aware of having been punished for their transgression and therefore that punishment cannot discourage the violator from repeating the transgression in future (Crockett et al., 2014).

So the claim that people are intrinsically motivated to punish norm violation is in good shape. However, the nativist view that intrinsic motivation to punish is due to a human-specific, norm-specific mechanism is incompatible with recent evidence. Experiments using naturalistic methods, rather than economic games, indicate that costly third-party punishment occurs infrequently in Western populations when no one is watching (Balliet et al., 2022; Hofmann et al., 2014, 2018; Kriss et al., 2016; Molho et al., 2020; Pedersen et al., 2018). Alongside ethnographic evidence that people in small-scale societies rarely deliver costly punishment unless they or their kin have suffered serious harm (Boehm, 2008; Ericksen & Horton, 1992), this suggests that behavior in economic games, in which the experimenter is always watching, overestimates the power of intrinsic motivation to punish norm violation by confounding it with the desire to please or impress the people conducting the study (Pedersen et al., 2018, 2020).

If costly third-party punishment rarely occurs among humans in the wild—outside economic games—the gap between humans and other animals is smaller than nativist norm psychologists assume. Dominant chimpanzees punish conspecifics who steal their food (Riedl et al., 2012), and many animals—including wasps, mole rats, and fairy wrens—respond aggressively to conspecifics that fail to show cooperative behavior (Clutton-Brock & Parker, 1995). It is not known how often or how much this retaliatory behavior costs the perpetrators, and therefore whether it meets a strict definition of costly punishment (Jensen, 2016; Raihani et al., 2012), but research on “appetitive aggression” (Rasa, 1976) suggests that it is intrinsically motivated—that the aggressors enjoy it. For example, studies of laboratory rodents—rats, mice, and Syrian hamsters—show that they will work for the opportunity to fight with a conspecific and develop a preference for places where they have fought in the past (Aleyasin et al., 2018).

### Summary of problems

Nativist norm psychology has encountered conceptual and empirical challenges. On the conceptual side, it has become evident that defining norms with reference to rules or standards breeds uncertainty about the explanatory target of norm psychology. In addition, it is not clear why the nativist view assumes that the universality of norms, along with their importance, early development, and intrinsic motivation, is a sign that norm processing must be done by innate, domain-specific mechanisms. On the empirical front, the nativist view is incompatible with evidence that adults and children conflate descriptive and prescriptive norms, that domain-general learning plays a significant role in norm acquisition, and that the social-learning biases modulating norm acquisition are either innate and domain-general or culturally learned and domain-specific rather than domain-specific and innate. The evidence remains strong that people are intrinsically motivated to punish norm violation, but recent work indicates that the mechanisms responsible are continuous with those found in nonhuman animals. These conceptual and empirical problems suggest that the field needs a new framework for norm psychology.

## A Cultural-Evolutionary Framework for Norm Psychology

**Fig. 2.**
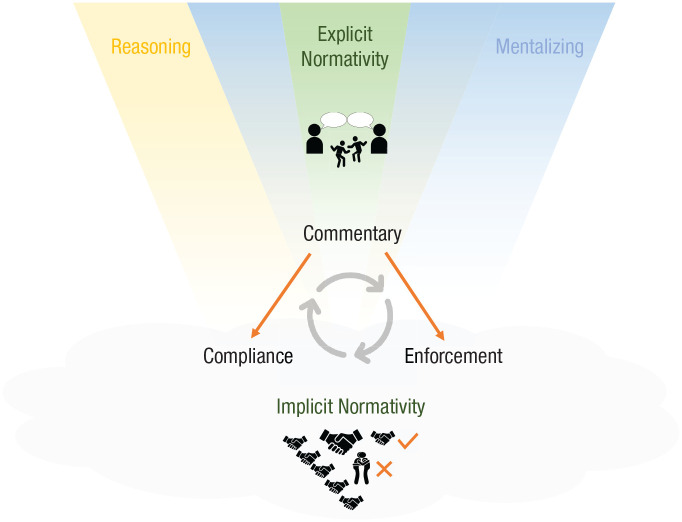
A cultural-evolutionary framework for norm psychology. Normativity (center) consists of three kinds of behavior: compliance, enforcement, and commentary. The actions that constitute compliance and enforcement in a society are defined relative to one another and by commentary in that society (straight arrows), not by their dependence on psychological processes specialized for norm processing. Implicit normativity (lower panel): In early childhood, commentary is absent, and compliance and enforcement depend exclusively on implicit, domain-general psychological processes found in a wide range of animals. Implicit acquisition processes are sensitive to frequencies and outcomes of observed and executed actions in various settings (greeting icons). Explicit normativity (upper panel): The development of explicit, domain-specific normative processes occurs gradually and is not complete until adulthood. Rooted in implicit compliance and enforcement and driven by cultural learning—primarily by adult and peer commentary (speech bubble icons)—the developmental process fashions the capacity for rule-based thought about what is appropriate, allowed, required, and forbidden (green) from reasoning (yellow) and mentalizing (blue). At the population level, the developmental process and the psychological mechanism it produces are shaped by cultural selection. Implicit-explicit relations (center): Explicit normativity enables commentary and influences compliance and enforcement by modulating the behavioral outputs of implicit processes (curved arrows).

In this section, I outline a new cognitive-evolutionary framework for norm psychology, proposing that in humans, normative competence depends on domain-general psychological processes plus a culturally evolved cognitive gadget (Fig. 2; Heyes, 2018a, 2019b).

Like nativist norm psychology, the cultural-evolutionary, or gadget, account assigns important roles to nature (genetic inheritance), nurture (learning), and culture (social inheritance) in the development of normativity, but the balance between them is different. The nativist view casts genetic evolution (natural selection acting on genetic variants) and the gadget view casts cultural evolution (natural selection acting on cultural variants) as the principal architect of norm psychology—the population-level process that has shaped norm psychology and made it broadly adaptive. The culture–gene coevolutionary hypothesis behind the nativist view suggests that socially inherited norm content was a crucial source of selection pressure for the genetic selection of normative thinking. Socially inherited norm content played a role that is filled by climatic and ecological variables in other evolutionary trajectories. In contrast, the gadget view suggests that it was selection among socially inherited variants that “designed” the special ways in which people think about norms. The cultural domain was not just the source of selection pressure but also the site of Darwinian selection.

As a corollary at the individual level rather than the population level, the nativist view suggests that humans genetically inherit Big Special psychological resources for norm processing, such as mechanisms specialized for the detection of norm-implicating behavior or to infer rules from such behavior. In contrast, the gadget view suggests that the human-specific, genetically inherited psychological resources that contribute to the development of norm processing are Small Ordinary. Genetic evolution has expanded people’s domain-general capacities to learn and remember (Fagot & Cook, 2006; Holland, 1992) and tweaked attentional and motivational processes—for example, giving people biases to attend to faces and voices (Johnson et al., 1991; Reid et al., 2017; Vouloumanos & Werker, 2007) and greater sensitivity to social rewards (Floccia et al., 1997)—making people much more receptive to social information than their hominin and other primate ancestors (Heyes, 2018a, Chapter 3). But on the gadget view, this genetic “starter kit” is not norm-specific (Frith, 2001; U. Heyes, 2018a). It helps people to learn from others about everything, not just about norms.

In a complementary way, the gadget view assigns a weightier role to social inheritance, to culture, in the development of normativity. In both the nativist and gadget frameworks, norm content is learned from other people. People learn that cousin marriage is prohibited or that hat wearing is required by verbal instruction, by observing the behavior of others in their social group, and through exposure to the rewards and punishments they deliver when people comply with or violate the group’s norms. However, on the gadget view, people also learn via these social routes—conversation, observation, and interaction—how to think and feel about norms in general. People learn from other people to represent norms as a distinctive kind of regularity in the social world and emotions—including guilt, shame, and righteous indignation—that motivate compliance with and enforcement of norms (L. F. Barrett, 2017; Hoemann et al., 2020).

To make comparison easier, the structure of this section, in which I outline the cultural-evolutionary account of norm psychology, mirrors the structure of the second section, in which I outlined the nativist-evolutionary account. I starts with a new characterization of norm psychology, focused on behavior rather than rules, that is designed to overcome the explanatory target problem. Then I address the problem of evidential relations with a poverty-wealth scheme for assessing evidence that a psychological capacity has been shaped by nature (or is innate), nurture, and culture. In the final part, I sketch a new psychological model of normativity.

### Characterization-behavior

Viewed as a natural phenomenon, norm psychology is the set of psychological processes responsible for normative behavior. Viewed as a research project, norm psychology aims to elucidate the psychological processes responsible for normative behavior. There are three types of normative behavior: (a) compliance—normative agents tend to behave in ways that are approved by other members of their social group and to avoid behaving in ways that are forbidden; (b) enforcement—normative agents tend to react positively to others when they behave in ways that are approved and to react negatively when they behave in ways that are forbidden; and (c) commentary—spontaneously and when questioned, human normative agents make judgments—they say things—about the types of behavior that are appropriate, allowed, required, and forbidden (i.e., about what ought and ought not to be done). These three kinds of normative behavior, or “normativity,” are closely related. For example, agents’ behavior counts as compliant, rather than merely conformist, to the extent that it is consistent with their group’s enforcement behavior and/or commentary (House et al., 2020).

Regardless of whether the nativist- or cultural-evolutionary account turns out to have more value, this behavior-based characterization is recommended as a way of integrating psychology with other disciplines investigating normativity. It foregrounds the traditional explanatory target of psychology—behavior—without moving too far away from the conceptions of norms used in anthropology, history, law, philosophy, and sociology. The behavior-based approach leaves room for people to think of norms as rules—spoken or written statements—or standards that have been inferred from compliance, enforcement, and commentary behavior by people in a society or by observers from outside. It also leaves room for evidence that mental rules—sentences in the head—generate compliance, enforcement, and commentary behavior, but the behavior-based approach does not prejudge this issue. It encourages people to think freely about the psychological mechanisms that could generate normative behavior not only in adult humans but also in children and nonhuman animals. In principle, they could be domain-specific, or domain-general, or a mixture of both; they could function as a system, with high levels of interdependence, or in a more piecemeal fashion. These are empirical questions.

### Evidence of nature, nurture, and culture

There is compelling evidence that normative behavior is culturally universal, important, early developing, and intrinsically motivated. However, it is not clear why nativist norm psychologists take this evidence to indicate that the processes responsible for normative behavior are innate and domain-specific (see above). To create a healthy research environment, in which alternative hypotheses can be tested against one another by evidence and argument, it would be helpful to have a scheme indicating the kinds of observations that count in favor of (and against) innateness, learning, and cultural inheritance of psychological characteristics.

I have argued that an expanded version of Chomsky’s (1965) poverty-of-the-stimulus argument provides such a scheme (Heyes, 2018a, 2019b; Ray & Heyes, 2011). This approach asks whether the developmental environment provides too little (poverty) or at least enough (wealth) usable information to explain the characteristics of a target psychological mechanism. Poverty is a sign that the development of a psychological trait depends on genetically inherited information (nature), whereas wealth is a sign that development depends on learning (nurture) and/or on culturally inherited information (culture). Both nurture and culture require learning, but in the nurture case, individuals learn through their own efforts about social or asocial features of their world, whereas in the culture case, they learn from others; what I learn by interaction with you depends not just on what you do but also on what you know (Heyes, 2019a). Consequently, where there is evidence of wealth, nurture is indicated if development varies with features of the environment in which development actually occurs (i.e., with information that can be acquired by asocial learning and by the kinds of social learning found in a broad range of animals). Culture is indicated when development varies with longer-term features of the environment; features that may not be present when a particular individual is developing or that can be acquired only via the kinds of distinctively human social learning known as cultural learning. Training studies can help to distinguish the roles of nature, nurture, and culture (e.g., De Klerk et al., 2015; Lohmann & Tomasello, 2003), but many of the empirical methods with the power to parse development in this way examine patterns of spontaneous covariation. They relate differences in cognitive ability to opportunities for learning, social learning, and cultural learning across (a) time points in development, (b) groups or individuals in a human society, (c) human societies, or (d) species (Heyes, 2018a, 2019b).

In this poverty-wealth scheme, evidence that a component of norm psychology is culturally universal (comparison across human societies) constitutes evidence of poverty of the stimulus, and therefore innateness, only if there is ancillary evidence that the universality could *not* be due to convergent learning and cultural evolution. Likewise, signs of early development (comparison across time points in development) count as evidence of innateness only if studies of the developmental environment indicate that children do not have the opportunity to acquire the normative characteristic via domain-general mechanisms or cultural learning before its emergence. The other two pillars of the nativist case, importance and intrinsic motivation, do not have an obvious place in the poverty-wealth evidential framework. Given that cultural evolution can produce important psychological characteristics (e.g., literacy) and that intrinsic motivation can be learned (see below), it is not clear why these characteristics of normativity have been seen as evidence of innate, domain-specific normative processing.

The poverty–wealth scheme suggests that two types of evidence have been underexploited by norm psychologists. Variation among groups and individuals in a society (b above) and across species (d above) could provide important clues to the origins of norm psychology but is rarely mentioned by individuals who identify as norm psychologists (Westra & Andrews, in press).

### Model

The cultural-evolutionary model suggests that two types of psychological mechanism support normative behavior: implicit and explicit. Research on decision-making and cognitive control indicates that implicit processes are typically fast, automatic, associative, effortless, and nonconscious. In contrast, explicit processes are typically slower, controlled, rule-based, effortful, and conscious. Explicit processes do tend to interfere with one another, and implicit processes do not (Evans & Stanovich, 2013a, 2013b; Norman & Shallice, 1986).

The implicit processes supporting early development of normative behavior are domain-general and taxon-general; they are perceptual, attentional, learning, memory, motoric, and motivation processes that support normative and nonnormative behavior via the same computations in a wide range of animal species. These implicit processes, although domain-general, could be described as innate. Their development is canalized and depends on genetically inherited information and experience (nature and nurture) but minimally or not at all on culturally inherited information (culture). The explicit processes are domain-specific. They represent the expectations of others in concepts—recombinable elements of conscious, deliberate thinking (Shea, 2018)—and language-like mental rules about what is appropriate, allowed, required, or forbidden in different contexts for various members of a social group. Most of these rules, and the capacity to reason with them in distinctive ways, are learned from other people via language and other forms of cultural learning.

The innate, domain-general implicit processes get normative behavior off the ground, phylogenetically and ontogenetically. They are solely responsible for compliance and enforcement in early childhood and continue to play important roles throughout adult life. However, explicit processes become increasingly influential in middle childhood. They support commentary behavior and influence both compliance and enforcement by modulating the behavioral outputs of implicit processing. Commentary behavior—statements about what is appropriate, allowed, required, and forbidden—has synchronic and diachronic functions. Within generations, commentary enables negotiation of norm content (e.g., attitudes to gay marriage) and debate about what constitutes norm violation (e.g., through gossip and legal processes; Jolly & Chang, 2021). Between generations, commentary facilitates cultural learning of norm content and, recursively, of the explicit normative processes that make commentary possible.

“Two-system” or “dual-process” theories, postulating implicit and explicit or “automatic” and “controlled” processes are common in moral psychology (Cowell et al., 2018; Crockett, 2013; Cushman, 2013, Greene, 2017, Rhodes & Wellman, 2017) but not in norm psychology (for an interesting exception, see Kelly, 2020a, 2020b). The following sections outline the ways that implicit and explicit processes give rise to normative behavior—compliance, enforcement, and commentary. They refer to psychological processes that are modeled in a variety of ways in contemporary cognitive science, for example, as associative or statistical learning, model-free or model-based reinforcement learning, mental models or pragmatic schemas, and assuming inverse or forward (“predictive”) processing. The cultural-evolutionary account of normativity is not committed to particular modeling strategies. Rather, it is committed to the view that mature human normativity is (a) rooted in implicit psychological processes that, however they are modeled, do many jobs in many species and (b) distinguished from nonhuman normativity by cognitive and motivational features that are culturally learned.

Implicit processes support the early development of compliance and enforcement behavior.

#### Compliance

In infancy and early childhood, compliance depends on domain-general processes of categorization and reinforcement learning (Ayub & Wagner, 2020; Foster-Hanson et al., 2021; Morris & Cushman, 2018; Schlegelmilch et al., 2021; Wellman et al., 2016). For example, in adult commentary, “giving” is more normatively loaded than “reaching,” but young children learn to categorize a variety of different body movements as (what an adult would call) “giving” and that giving has positive outcomes in many contexts (e.g., hugs) in the same way that they learn to treat a variety of different body movements as “reaching” and that reaching has positive outcomes in many contexts (e.g., toys; Pulverman et al., 2006). Direct social rewards, such as receiving a hug or a smile, are especially important when learning compliance, but direct experience is augmented by observation of others’ behavior in both normative and nonnormative cases (Haaker et al., 2021). Young children can learn when to give by watching another child give a toy and get a hug, and they can learn via the same process to reach by watching another child reach and secure a toy. Social learning of this kind, which also occurs in rats (Saggerson & Honey, 2006) and birds (Heyes & Saggerson, 2002; Saggerson et al., 2005), is made possible by “conditioned” or “secondary” reinforcement (Williams, 1994). The sight of another agent’s action acquires positive or negative value for the observer when it has been positively correlated with similar, direct experience—for example, when the learner has eaten while seeing others eat or felt distress while observing the distress of others (Heyes, 2018b).

Once established, compliant behavior may be maintained by habit. For example, children in Victorian society may have learned to stand whenever an adult entered a room by tracking social rewards and punishments, but with repetition, standing became a reflex response to adult arrival (Adams, 1982; Pool et al., 2022). It would have been difficult for them to stop if they had been told not to do it. Compliant behavior can also be maintained by a type of conditioned reinforcement that amounts to intrinsic motivation (Kruglanski et al., 2018). During the acquisition of a compliant behavior, the feeling of doing the action—for example, the sensations from her own body and from the world that a child feels when she rises from a seated to a standing position—are repeatedly correlated with extrinsic rewards, attention and smiles from others, in a distinctive setting, the arrival of an adult. Consequently, the feeling of doing—the stimulus change resulting from action execution—becomes rewarding in that setting (Osborne, 1977). The child is no longer motivated by the expectation that she will get attention or other extrinsic rewards. She enjoys performing the action for its own sake.^5^

#### Enforcement

Early enforcement builds on early compliance and, like early compliance, depends on domain-general processes (Seymour et al., 2007). Research on the conflation of descriptive and prescriptive norms (see above) has shown that behaviors receiving normative approval in adult commentary are more common and therefore, on average, more familiar and predictable than behaviors that are not approved. Decades of research on the “mere exposure effect” shows that humans and other animals like familiar stimuli—images, sounds, tastes, and smells they have encountered without reinforcement—more than unfamiliar stimuli (Bornstein & Craver-Lemley, 2016). Conversely, prediction error, produced by the occurrence of unexpected events, is associated with increased arousal that is typically aversive (Theriault et al., 2021). Therefore, much enforcement behavior, in children and throughout life, is likely to be very simple indeed. An individual observing familiar, normative behavior feels good and is therefore more likely to be friendly toward the actor, whereas an individual observing unfamiliar, nonnormative behavior feels bad and is therefore more likely to be unfriendly. Evidence of this pattern comes from research showing that when emotional states are induced by unrelated videos, happy people donate more and punish less than angry people (Drouvelis & Grosskopf, 2016). In addition, studies of aversion-induced aggression indicate that humans, like a wide range of other animals (e.g., hamsters, gophers, monkeys, cats, chickens, snakes, and turtles), respond aggressively not only when provoked by the behavior of another agent (Clutton-Brock & Parker, 1995) but also—underlining the domain-generality of the underlying mechanisms—when in pain, stressed, or uncomfortably warm (Groves & Anderson, 2018). This kind of “reactive aggression”—in contrast with goal-directed, “proactive aggression”—is a highly conserved trait (Wrangham, 2018).

The effect of familiarity-based enforcement behavior is to reward the normative behavior of others and to punish their counternormative behavior. That is what makes it “enforcement.” However, the implicit processes mediating familiarity-based enforcement do not categorize observed action as normative or nonnormative. They just register how often and how recently the behavior has been observed under similar circumstances in the past. Furthermore, these implicit processes do not represent the potential effects of the enforcer’s behavior on the target. The enforcer does not act with the intention of rewarding, punishing, or having any effect at all on the target’s behavior. In this sense, familiarity-based enforcement is intrinsically rather than instrumentally motivated.

Of course, reinforcement learning, in which outcomes are important, also contributes to early enforcement. It would be strange if infants could learn via reinforcement mechanisms to control the movements of toys (Kenward et al., 2009) and videos (Klossek et al., 2008) but not the movements of other people. It would be odd if, for example, infants could not learn that creating a physical obstruction or saying “Stop!” can make another actor stop doing something unpleasantly novel and start doing something that is pleasingly familiar or an action that is associated in the enforcer’s mind with positive extrinsic rewards. When adult commentary would class the unpleasantly novel behavior as “bad” or “wrong” and the alternative as “good” or “right,” the enforcing child may also get a warm response (social reward) from an observing adult, making her yet more inclined to enforce familiarity in future.

Recent research on “normative protest” can be used to illustrate how implicit processes yield early norm enforcement. This fascinating work suggests that children begin to show enforcement when they are 18 months (Schmidt et al., 2019) or 3 years old (Schmidt et al., 2016). Children at these ages observe an adult model performing an action on an object and are then either given access to the object themselves or allowed to observe a puppet moving the object in the same way as the adult or in a different way. When children manipulate the object themselves, they typically repeat the adult’s action. When the puppet moves the object in a different way, the children often intervene by putting the object in the location where it was placed by the adult or saying things like “No!,” “Stop!,” or “Must go in there!” (Schmidt et al., 2019).

On an implicit-processing account, the children given access to the object copy the model because that yields a more familiar outcome. They may have seen the action only once, but it was just seconds or minutes ago. In addition, they are inclined to copy an adult because copying has been rewarded in similar contexts in the past—for example, when an adult behaves without hesitation in full view of the child. In other words, they copy in the expectation of a good outcome—social approval or a warm feeling because of conditioned reinforcement (Miller & Dollard, 1941; Schein, 1954). On an explicit-processing account, rooted in nativist norm psychology or another nativist framework, the children copy because they have a genetically inherited drive to act in the same way as others in their social group (Hoehl et al., 2019). The implicit and explicit accounts could be tested against one another by, for example, varying the children’s experiences of reward for copying. If domain-general implicit processes are responsible, children should learn readily via contingent reward to copy young models, members of out-groups, or people who appear to have moved an object carelessly. If domain-specific explicit processes are responsible, this kind of learning should be difficult or impossible (Jagiello et al., 2022).

On an implicit-processing account, children protest when the puppet moves the object in a different way from the model because they experience aversive prediction error and anticipate omission of expected reward—that they will miss out on the approval or warm feeling. Consequently, the protestors behave in an unfriendly way toward the puppet and intervene to restore familiarity. Their utterances are instrumental responses. By 18 to 36 months, some children have discovered via reinforcement learning that “No!,” “Stop!,” and “must” are “pushy words,” words that are apt to change the behavior of another agent. On an explicit-processing account, the children’s utterances are commentary behavior, rooted in explicit normative rules, reflecting some understanding of what is expected in the social group and approved by its members. This is possible but unlikely given that the children in these experiments are given no evidence that the adult’s behavior is descriptively or prescriptively normal. They see the behavior performed once, by one person in one context, and without affirmation. If their utterances are commentary, their normativity is perverted rather than “promiscuous” (Schmidt et al., 2016), untethered from its proper domain. Nonetheless, the implicit and explicit accounts of protest could be tested against one another by, for example, equating the familiarity of the puppet’s “normative” and “counternormative” actions or by selectively rewarding the children for doing the opposite of an adult’s action before the focal experiment. If implicit processing is responsible for protest, protest should decline as the familiarity of the alternative action increases and flip when alternative action has been rewarded; children should protest when the puppet performs the same action as the model rather than a different action. If explicit processing is responsible, these manipulations should have little effect.

Pending dedicated experiments to test the implicit and explicit accounts, the implicit interpretation is consistent with electroencephalographic data from a study in which preschool children observed an adult performing a counternormative behavior (ripping a page out of a library book). When looking at pictures of harming, the children who protested the adult’s transgression differed from children who did not protest on an early event-related-potential component (central P2) that indexes perceptual sensitivity and sustained attention, described as “implicit moral evaluation” (Kim et al., 2021).

Thus, implicit normativity is intrinsically and extrinsically (or instrumentally) motivated, and its development depends in part on simple forms of social learning—on vicarious and direct experience of rewards and punishments delivered by other agents. The implicit processes outlined above are thoroughly normative in that they typically bring the agent’s behavior (compliance) and the behavior of their social partners (enforcement) into conformity with one another and with what is approved by normative commentary in the agent’s society. But they are not dedicated to that function. In other contexts, the same implicit processes do completely different jobs—for example, enabling the development of food preferences and the acquisition of motor skills. Furthermore, implicit processes are not rule-based in any interesting sense. They are not necessarily or exclusively based on stored exemplars of behavior (the criterion of Sripada & Stich, 2006), and the behavioral regularities they produce can be described by rules, such as “Children should stand when an adult enters a room,” but these are very low bars for rule-hood. Even the learning of simple categories, such as “bird,” does not depend necessarily or exclusively on stored exemplars (Schlegelmilch et al., 2021), and any regularity in nature, anything that is not random, can be described from the outside by a rule. Implicit normativity does not have rules on the inside; it is not produced by rule-like mental structures, by sentences in the head.

#### Explicit processes

Explicit normativity begins to augment and interact with implicit normativity when children start to represent not only what others do but also what others expect to be done (Bicchieri, 2005; Theriault et al., 2021). As explicit processes emerge, a child who has learned to share toys in anticipation that sharing will make other people do rewarding things, such as smile, begins to appreciate that the people around them expect sharing in some contexts and that their reactions to the child’s behavior in these contexts—smiling, grabbing, reprimanding—depend on their expectations. Likewise, a child who previously repeated adult actions only because the repetition yielded pleasant feelings of familiarity or because it made other people smile begins to see repetition as expected and social rewards and punishments as contingent on this expectation.

The explicit processes that contribute to normative behavior are often labeled “deontic reasoning,” a distinctive type of deductive reasoning in which information about what is appropriate, allowed, required, or forbidden is represented by proposition-like “mental models” (Ragni et al., 2018) or “pragmatic schemas” (Holyoak & Cheng, 1995). In contemporary cognitive science, models of deontic reasoning are pitched at a high level of abstraction (Beller, 2010). They capture core semantic and syntactic features of the explicit processes but rarely acknowledge that the rules of deontic reasoning represent social facts, the expectations of others, and therefore depend on “social understanding” (Carpendale & Lewis, 2015).

Some processes of social understanding, known as “explicit mentalizing,” “mindreading,” or “theory of mind,” represent mental states. They represent an expectation as something inside an individual’s head that arises from the individual’s experience and influences their behavior. Other explicit processes of social understanding encode behavioral rules, such as “Drive on the right” and “Help members of your group,” and the situations in which these rules apply. They represent an expectation as something that resides in a group, situation, or institution rather than in the minds of individual agents. Cross-cultural evidence suggests that both kinds of explicit process contribute to normativity in all human societies, but cultures vary widely in the extent to which they rely on mental-state attribution rather than behavioral rules (Taumoepeau, 2019). For example, 38% of 12- to 14-year-old ni-Vanuatu children from Nguna Island “fail” a classic false-belief test of mentalizing that is typically “passed” by 4- to 5-year-olds in the United States (Dixson et al., 2018). In a complementary way, Japanese children given the same test—in which children are asked to predict where a protagonist will look for an object that was moved in their absence—are more likely than children in Europe and North America to explain their prediction with reference to behavioral rules and situational factors rather than mental states (Naito & Koyama, 2006).

The greater reliance of “interdependent” or “relational” cultures on behavioral rules suggests that in these societies, explicit normativity depends more heavily on rules than in “individualistic” cultures. For example, the helping behavior of a person of European heritage may be more likely than that of a Japanese person to spring from an explicit but amorphous belief that others expect helping—a belief rooted in that person’s prior experience in similar circumstances but not encoded in an explicit, sentence-like mental structure specifying what is appropriate, allowed, required, or forbidden. This is consistent with evidence from Western samples that a good deal of people’s normative commentary, of their statements about what is and is not right, is post hoc rationalization. People can usually formulate rules and engage in deontic reasoning when questioned, but their normative behavior is generated by implicit processes (Haidt, 2001, 2012).

There is evidence that explicit normativity is not “in our genes.” This evidence indicates wealth of the stimulus and implicates cultural learning—human-specific forms of social learning—in the development of explicit normativity (Dunn, 1994; Dunn & Hughes, 2014; Grusec, 2014; Grusec et al., 2014; Wright & Bartsch, 2008). For example, children in China, a relational culture, show indirect reciprocity earlier than children in Germany, an individualistic culture, at 3 rather than 5 years of age (Li et al., 2020). In Western samples, there is a positive correlation between the number of auxiliary modal verbs used by children and the number used by their parents (Wells, 1979). Six-year-old children, who usually cannot extract prescriptive messages from stories, become able to do so when they explain the story to an adult (C. M. Walker & Lombrozo, 2017). The normative development of 10- to 15-year-olds, measured by their comments on a series of moral dilemmas, is predicted by the quality of their spontaneous conversation with parents and peers about normative questions. A “Socratic style of eliciting the other’s opinion and checking for understanding” and conversational focus on the child’s experience of moral conflict are especially effective in promoting normative development (L. J. Walker et al., 2000, p. 1045). From 5 to 86 years of age, normative development measured by Kohlberg’s tests of moral reasoning (Colby et al., 1987) is linearly related to number of years of formal education (Dawson, 2002).

Evidence of this kind suggests that “ought thought” (Westra & Andrews, in press)—explicit psychological processes specialized for normativity—is concocted from more domain-general mentalizing and reasoning processes through conversation, observation, and interaction in the course of development (Cushman et al., 2013; Hawkins et al., 2019; Ho et al., 2017; Imuta et al., 2016; Rhodes & Wellman, 2017; Sterelny, 2021). The explicit processes of deontic reasoning used by some minorities, for example, lawyers, ethicists, and moral philosophers in Western societies, have been constructed by “intelligent design” (Dennett, 2009); like algebra or calculus, they have been fashioned deliberately by generations of scholars with the express purpose of finding the right way of thinking about what is right. Other people use explicit normative processes that have been cobbled together primarily by cultural selection. Insofar as these processes do the jobs traditionally associated with normativity—promoting cooperation and true values—it is because groups with an explicit norm psychology that did those jobs relatively well passed on their explicit norm psychology, via cultural learning, to a larger number of descendants (Birch & Heyes, 2021).

#### Implicit–explicit relations

Implicit normativity provides a foundation for the development of explicit normativity (Rhodes & Wellman, 2017). A child without a repertoire of compliance and enforcement behavior acquired by implicit processes would struggle to get the message about others’ normative expectations. Even if she developed mentalizing and reasoning, it is unlikely they would coalesce into culture-typical explicit normativity because there would be little of personal relevance to be explained by the expectations of others. A rule about helping makes sense not only of what a child sees others doing but also of her own implicit feelings and behavior. It explains (or rationalizes) why she sometimes helps when she does not want to and the uneasy feeling when she fails to help and, pleasingly, casts her feelings when others fail to help as righteous indignation. Explicit processes interpret interoceptive experiences. They transform feelings of variable intensity (arousal) that are merely good or bad (valence) into full-blown emotions such as shame, guilt, and moral rage—the intrinsic motivators of explicit normativity (L. F. Barrett, 2017; Theriault et al., 2021). Guilt and shame are forms of self-punishment that make one’s compliance less dependent on others’ enforcement (C. Frith & Frith, in press).

In some cases, the acquisition of specialized, explicit cognitive processes has profound effects on domain-general implicit processes. For example, learning algebra changes perception (Marghetis et al., 2016). The extent to which explicit normativity transforms implicit normativity remains a contentious empirical question. In principle, explicit processes could take over entirely or do nothing more than support normative commentary, providing post hoc justification for actions and judgments caused by implicit processes (Haidt, 2001, 2012). Not surprisingly, current evidence suggests an intermediate reality in which explicit normative processes augment and regulate but do not replace implicit normative processes in generating compliance and enforcement behavior. Evidence of regulation comes from the behavior of adults playing economic trust games, in which explicit processes, activated by instructions about another player’s reputation or moral character, suppress subsequent reinforcement learning (Delgado et al., 2005; Fouragnan et al., 2013). Likewise, developmental research indicates that from 6 years of age in Western samples, explicit or “controlled” normative processes can mediate sharing (compliance), costly punishment (enforcement), and intent-based normative judgment (commentary; Chernyak & Kushnir, 2018; Cushman et al., 2013; McAuliffe et al., 2015; Steinbeis, 2018).

Further evidence of the top-down influence of explicit processes comes from studies manipulating belief in free will (C. Frith & Frith, in press), a normatively charged belief with marked cultural variation (Berniu-nas et al., 2021). Laboratory studies with Western samples show that people who have been induced to doubt the existence of free will are less helpful and more aggressive (Baumeister et al., 2009), more likely to cheat in exams (Vohs & Schooler, 2008), and, crucially, less likely to show error slowing after an error in a simple reaction-time task (Rigoni et al., 2013). The effects of the belief manipulation on helping, aggression, and cheating could be due to explicit normative thinking alone, but error slowing after an error is due to a paradigmatically implicit process (Dutilh et al., 2012).

There is also evidence that implicit, domain-general mechanisms of categorization and learning continue to have an unregulated or minimally regulated effect in adult life (Bear & Knobe, 2017; Burton-Chellew & Guérin, 2021). For example, people playing economic games are more likely to show prosocial giving behavior when they have been exposed, in the lab or their ordinary lives, to institutions that are effective in punishing behavior that deviates from giving norms. However, exposure to these institutions does not make people more likely to punish other players who violate giving norms. This suggests that institutions have narrow effects, the kind one would expect if they produce behavior change via implicit reinforcement learning. If institutions were causing conceptual change—for example, development of an explicit belief that “giving is right”—one would expect them to influence both giving behavior and punishment of people who fail to give (Stagnaro et al., 2017).

Explicit processes can also become implicit. Like driving a car, patterns of normative thought that were once deliberative—conscious, effortful—can become automatic with intensive practice (Pizarro & Bloom, 2003). Rules such as “Do not tamper with nature” and “Acts are worse than omissions” can “go underground,” becoming “heuristics” or “intuitions” with a pervasive influence on behavior that is unexplained by (“moral dumbfounding”; Haidt, 2001) or at odds with the actor’s normative commentary (Sunstein, 2005). Unlike the implicit processes that initiate normative development, these heuristics are domain-specific—they apply only to what one ought and ought not to do—but their origins lie not in the genes, but in a socially inherited apparatus of normative thinking.

## Conclusion

### Objections

The cultural-evolutionary framework suggests that human normative competence depends on domain-general psychological processes plus a cognitive gadget—a domain-specific way of processing norms shaped by cultural selection (Heyes, 2018a, 2019b). A common and potentially powerful objection to cognitive gadgets says that they would have become cognitive instincts (e.g., Del Giudice, 2019; Dor et al., 2019; Turner & Walmsley, 2021). Even if distinctively human cognitive mechanisms were at first socially inherited and shaped by cultural selection, a process variously known as “Baldwinisation” (Baldwin, 1896), “canalization” (Gottlieb, 1991), and “genetic assimilation” (Waddington, 1953) would have favored genetic variants that reduced the environmental input necessary for their development. In this way, the erstwhile gadget would be subjected to genetic selection and, over many generations, become innate—genetically rather than culturally inherited.

Plausibility arguments and modeling are not sufficient to establish that Baldwinization is likely to have affected gadgets in general or the norm gadget in particular. It is plausible that normative behavior has been important to human survival and reproduction for long enough, measured in biological generations, for the Baldwinization of norm-specific cognitive mechanisms. However, it is also plausible that social inheritance of norm-specific mechanisms was cheap and reliable enough that genetic mutations with the potential to reduce environmental input conferred little or no selective advantage (Morgan & et al, 2020). A more promising approach, pursued incisively by Turner and Walmsley (2021), looks to empirical research on “preparedness” for evidence of Baldwinization. This research is widely believed to show that taste aversion (Garcia & Koelling, 1966) and fear learning (Seligman, 1970) depend on genetically evolved, domain-specific learning mechanisms—processes that forge associations via distinctive computations when, for example, a “fear-relevant” object such as a snake, rather than a “fear-irrelevant” object such as a flower, is experienced with an aversive stimulus (H. C. Barrett & Broesch, 2012). In principle, any domain-specific features of prepared learning could be due to standard or “aplastic” (Morgan & et al, 2020) genetic evolution, but studies of experimental evolution in *Drosophila* indicate that preparedness is more likely to be due to Baldwinization (Mery & Kawecki, 2002).

Empirical work on preparedness is exactly the right place to look for evidence that cognitive gadgets would have been Baldwinized, but it does not deliver. Careful scrutiny of 50 years of research on preparedness confirms that taste aversion and fear learning are “special” but also indicates that genetic evolution has improved their efficiency only by tweaking perceptual and motor processes and their attentional and motivational modulators (Heyes et al., 2020). For example, fear-relevant stimuli such as snakes are genetically primed to attract attention (Gray & McNaughton, 2003; Thrasher & LoBue, 2016). This gives them privileged access to learning mechanisms, but those mechanisms use the same computations to learn about snakes and flowers. Contrary to early claims (Hugdahl & Öhman, 1977), fear learning extinguishes at the same rate as learning about fear-irrelevant stimuli (Åhs et al., 2018) and is equally malleable by instructions (McNally, 1987, 2016). Evidence of this kind—relating to fear learning, taste-aversion learning, language, and imitation—indicates Baldwinization of input and output devices, analogues of scanner and printer interfaces, not of core inference processes. Cognitive gadgets are core inference processes. Therefore, research on preparedness does not support the objection that cognitive gadgets would have been Baldwinized. On the contrary, it supports the idea that genetic selection provided a starter kit for the evolution of human minds not by fashioning Big Special cognitive mechanisms, such as dedicated processes for learning and implementing norms, but by tweaking input and output processes, such as social tolerance and motivation (Heyes, 2018a, 2019b).

### Implications

There are three key contrasts between the cultural-evolutionary and nativist accounts of norm psychology. On the gadget account, first, the heavy lifting is done by domain-general rather than norm-specific processes. Second, these domain-general, implicit processes track the frequencies and outcomes of behavior; they do not represent what others expect or what is allowed as mental rules or in any other way. Third, explicit processes are rule-based and norm-specific but culturally rather than genetically inherited.

To find out which account is closer to the truth and in what ways, more empirical work of two kinds is needed. First, to establish whether the early development of normative behavior is guided by the maturation of domain-specific or domain-general processes, more experiments that test these alternative hypotheses against one another are needed. At present, developmental studies by nativist norm psychologists typically test one rich, domain-specific hypothesis against another rather than against a leaner, domain-general alternative. For example, they ask whether infants want to help an adult or to engage with the adult, not whether the children (or more precisely, their neurocognitive system) is working to restore the predictability of their environment. On the other hand, domain-generalists often rely on modeling to show that normative jobs could be done efficiently by processes that are not specialized for the task. This kind of modeling is valuable, but to move from “how possibly” to “how actually,” researchers need data.

Second, to discover to what extent explicit normativity is culturally rather than genetically inherited, more research guided by the poverty-wealth scheme is needed. While nativist norm psychology was the only evolutionary framework available, individuals pursuing a cognitive-evolutionary account of normativity hardly needed to appeal to the poverty of the stimulus. In most cases, it was obvious that children would not achieve mature normative competence if they had to work it all out for themselves. Now there is a cultural-evolutionary alternative to the nativist view—a framework acknowledging the need for inheritance of normative thinking but arguing that the inheritance is social—it is apparent that evidence of universality, importance, early development, and intrinsic motivation is a blunt instrument. It implicates inheritance, but it does not indicate what kind of inheritance, genetic or cultural, is important. Research guided by the poverty-wealth scheme would chart more fully variation in normative reasoning across time points in childhood development, groups or individuals in a society, human societies, and species and, crucially, trace the sources of this variation to genetic factors and to opportunities for learning, social learning, and cultural learning. At present, there is a lingering assumption that normative reasoning shows quantitative but not qualitative variation; that some individuals and societies are better at it than others, but normative concepts, such as “obligation,” always have the same functional roles (Beller, 2010). Likewise, it is assumed that across individuals and cultures, normative reasoning depends on mental models *or* pragmatic schemas and involves a standard blend of mentalizing and behavior rules. If one lets go of these assumptions—rooted in nativist norm psychology and the view that Western, educated, industrialized, rich, and democratic (Henrich, 2020) societies are psychologically representative of all humanity—the cultural-evolutionary account predicts that one will find rich variation not just in what people believe is right but also in the explicit processes they use to think about rightness. Tracing this variation to its sources, a crucial step in distinguishing genetic from cultural inheritance (Sterelny, 2021), would involve a new kind of behavioral genetics that charts experiential inputs to development, opportunities for social and cultural learning, just as carefully as genomic inputs.

What about normativity in the wild? The cultural-evolutionary account implies that normative behavior is less constrained and less secure than the nativist view suggests. The implicit processes generating compliance and enforcement in early development and throughout people’s lives did not evolve specifically to promote cooperative behavior. Therefore, they are not constrained genetically to produce behavior that, on average and over long periods of time, is advantageous for the individual or that has prosocial effects at the population level. Acting on implicit processes alone, people will comply with and enforce any behavior that is common and has yielded positive outcomes in their recent experience. It does not matter whether, in the long term, the behavior promotes or interferes with their own well-being or the thriving of their narrow social group—the people with whom they interact—or of the wider society in which they live. Implicit processes also allow people’s compliance and enforcement behavior to change relatively rapidly with experience. Habit formation and intrinsic motivation create some inertia, but there is no equivalent among implicit processes of Sripada and Stich’s (2006) norm database (Fig. 1), in which normative rules are safely insulated from change after acquisition. To the extent that normativity depends on implicit processes, acquisition and implementation are continuous in two respects: They are not distinct processes, and the representations mediating normative behavior are continually subject to revision by new experience.

Because the implicit processes are minimally constrained by nature, there is more pressure on specialized, explicit normative processes to produce and maintain “good” behavior. However, the proper development of these processes is less assured on the cultural-evolutionary than on the nativist account. Instead of being programmed in people’s genes, the development of explicit normative thought depends on social practices and institutions that can change rapidly and radically with political and economic conditions. Furthermore, the evidence from science and everyday life that moral judgments often depend on “emotional” or “Type 1” processes (Haidt, 2001, 2012) suggests that even when explicit normativity has developed in a group-typical way, it often struggles to get a grip on behavior. In short, the cultural-evolutionary account implies that contemporary individuals and societies—parents, peers, educators, elders, politicians, and lawyers—have more responsibility than the nativist view implies. People’s actions do not just shape and transmit the rules; they create in each new generation mental processes that can grasp the rules and put them into action.

## Transparency

*Action Editor:* Tina M. Lowrey

*Editor:* Klaus Fiedler

## Notes

1. In contrast with other cognitive gadgets—including imitation, mentalizing (also known as “mindreading” and “theory of mind”), and metacognition (Heyes et al., 2020)—motivational processes are important ingredients of the normativity gadget. It could be called a “cognitive-motivational” gadget.

2. It has been argued that the concept of innateness is misleading and outdated (Linquist, 2018), but it is used frequently by Sripada and Stich (2006) and other nativist norm psychologists (e.g., Henrich & Muthukrishna, 2021). Consequently, I use the term “innate” in this article, when reporting and commenting on the views of nativist norm psychologists, to refer to a trait that is predominantly genetically rather than socially inherited.

3. According to Sripada and Stich (2006), the explicit system may be dedicated to moral rules of the sort that have preoccupied philosophers and some developmental psychologists (e.g., Kohlberg, 1981), or it may be domain-general in the sense of dealing with factual as well as normative rules.

4. Long before “norm psychology” was born, Cosmides and Tooby (1989, 1992) carved a nativist path to norms with their research on “cheater detection” and “social exchange reasoning.” Curiously, this work is rarely cited by individuals who now identify as norm psychologists. This may be because there is a broad consensus among psychologists who study reasoning, “evolutionary psychologists” and others, that an innate module for cheater detection cannot satisfactorily explain what it was originally designed to explain—why people perform better on some versions of the Wason selection task than on others (Beller, 2010; Kaufman et al., 2011; Macchi et al., 2020; Manktelow & Over, 1990; Ragni et al., 2018; Sperber & Girotto, 2002; Valiña & Martín, 2016).

5. The idea that compliance in infancy and early childhood is due to implicit, domain-general processes is consistent with recent evidence that early helping is motivated by a desire to engage with other people rather than to promote their welfare (Carpendale et al., 2015; Pletti et al., 2017).
